# Comment on “Artificial Intelligence‐Enhanced Electrocardiography for Predicting Paroxysmal Atrial Fibrillation From Sinus Rhythm”

**DOI:** 10.1111/anec.70181

**Published:** 2026-03-25

**Authors:** Hadi Verdiyev, Bülent Görenek

**Affiliations:** ^1^ Department of Cardiology Private Rami Hospital Istanbul Türkiye; ^2^ Department of Cardiology, Faculty of Medicine Eskisehir Osmangazi University Eskisehir Turkey

To the Editor,

We read with great interest the recent article entitled “Artificial Intelligence‐Enhanced Electrocardiography for Predicting Paroxysmal Atrial Fibrillation From Sinus Rhythm: Impact of Data Integration Across Institutions and Devices,” published in *Annals of Noninvasive Electrocardiology*. The application of artificial intelligence (AI) to standard electrocardiograms (ECGs) recorded during sinus rhythm to identify individuals at risk of atrial fibrillation (AF) represents a rapidly evolving and highly promising field. Such approaches have the potential to improve early detection and screening strategies for AF. In this regard, the authors' effort to integrate datasets obtained from different institutions and devices is particularly valuable, as it may enhance the generalizability of AI‐based models. Nevertheless, several methodological aspects of the study merit further clarification.

First, the time window used to define AF‐positive cases deserves additional consideration. In the analysis, patients who developed AF within 31 days after an ECG recorded during sinus rhythm were classified as AF‐positive. However, the rationale for selecting this specific interval is not clearly explained. In patients diagnosed with AF within such a short timeframe, it is conceivable that ECGs recorded during sinus rhythm may already reflect subtle electrical changes associated with subclinical AF that has not yet been clinically recognized. Consequently, the algorithm may be detecting imminent or previously unrecognized AF rather than predicting the development of future AF. This distinction has increasingly been highlighted as an important methodological issue in AI‐based ECG research (Attia et al. 2019; Raghunath et al. 2021).

Second, the methods used to establish AF diagnosis are not described in sufficient detail. It remains unclear whether systematic rhythm monitoring strategies, such as prolonged Holter monitoring or implantable loop recorders, were employed during follow‐up. If AF diagnoses were based primarily on routine clinical encounters, some episodes of paroxysmal AF may have gone undetected. As a result, patients with undiagnosed AF might have been included in the AF‐negative group, potentially introducing bias into the labeling of the training dataset. Previous studies have demonstrated that a substantial proportion of paroxysmal AF episodes may be asymptomatic and therefore remain unrecognized during routine clinical evaluations (Xiong et al. 2015).

Another aspect worth considering is that the model was developed exclusively using ECG signal data. However, numerous clinical factors are known to influence AF risk, including age, hypertension, heart failure, and structural cardiac changes such as left atrial enlargement. Evaluating AI‐ECG performance alongside clinical variables, or developing combined prediction models, may help clarify the incremental value of the algorithm beyond conventional risk assessment.

Finally, the predictive performance of the AI model was not directly compared with established clinical AF risk scores, such as CHARGE‐AF, Framingham AF, or C2HEST (Alonso et al. 2013; Li et al. 2019). Such comparisons could provide useful insight into whether AI‐based ECG analysis offers meaningful improvement over currently available risk stratification tools.

In conclusion, the study provides valuable insights into the potential role of AI‐enhanced ECG analysis in AF risk prediction. Further clarification regarding AF labeling strategies, diagnostic methods, and comparisons with established clinical risk models may help better define the clinical utility of these algorithms.

## Author Contributions

**Hadi Verdiyev:** conceptualization, literature review, drafting of the manuscript, critical revision, and final approval. **Bülent Görenek:** conceptualization, supervision, critical revision of the manuscript, and final approval.

## Conflicts of Interest

The authors declare no conflicts of interest.

## Data Availability

The data that support the findings of this study are available from the corresponding author upon reasonable request.

## References

[anec70181-bib-0001] Alonso, A. , B. P. Krijthe , T. Aspelund , et al. 2013. “Simple Risk Model Predicts Incidence of Atrial Fibrillation in a Racially and Geographically Diverse Population: The CHARGE‐AF Consortium.” Journal of the American Heart Association 2, no. 2: e000102. 10.1161/JAHA.112.000102. 23537808 PMC3647274

[anec70181-bib-0002] Attia, Z. I. , P. A. Noseworthy , F. Lopez‐Jimenez , et al. 2019. “An Artificial Intelligence‐Enabled ECG Algorithm for the Identification of Patients With Atrial Fibrillation During Sinus Rhythm: A Retrospective Analysis of Outcome Prediction.” Lancet 394, no. 10201: 861–867. 10.1016/S0140-6736(19)31721-0. 31378392

[anec70181-bib-0003] Li, Y. G. , D. Pastori , A. Farcomeni , et al. 2019. “A Simple Clinical Risk Score (C_2_HEST) for Predicting Incident Atrial Fibrillation in Asian Subjects: Derivation in 471,446 Chinese Subjects, With Internal Validation and External Application in 451,199 Korean Subjects.” Chest 155, no. 3: 510–518. 10.1016/j.chest.2018.09.011. 30292759 PMC6437029

[anec70181-bib-0004] Raghunath, S. , J. M. Pfeifer , A. E. Ulloa‐Cerna , et al. 2021. “Deep Neural Networks Can Predict New‐Onset Atrial Fibrillation From the 12‐Lead ECG and Help Identify Those at Risk of Atrial Fibrillation‐Related Stroke.” Circulation 143, no. 13: 30–1298. 10.1161/CIRCULATIONAHA.120.047829. PMC7996054 33588584

[anec70181-bib-0005] Xiong, Q. , M. Proietti , K. Senoo , and G. Y. Lip . 2015. “Asymptomatic Versus Symptomatic Atrial Fibrillation: A Systematic Review of Age/Gender Differences and Cardiovascular Outcomes.” International Journal of Cardiology 191: 15–17. 10.1016/j.ijcard.2015.05.011. 25974193

